# Application of resequencing to rice genomics, functional genomics and evolutionary analysis

**DOI:** 10.1186/s12284-014-0004-7

**Published:** 2014-07-08

**Authors:** Longbiao Guo, Zhenyu Gao, Qian Qian

**Affiliations:** 1 State Key Laboratory of Rice Biology, China National Rice Research Institute, Chinese Academy of Agricultural Sciences, Hangzhou 310006, China

**Keywords:** Rice, Resequencing, Genomics, Functional genomics, Evolutionary analysis

## Abstract

Rice is a model system used for crop genomics studies. The completion of the rice genome draft sequences in 2002 not only accelerated functional genome studies, but also initiated a new era of resequencing rice genomes. Based on the reference genome in rice, next-generation sequencing (NGS) using the high-throughput sequencing system can efficiently accomplish whole genome resequencing of various genetic populations and diverse germplasm resources. Resequencing technology has been effectively utilized in evolutionary analysis, rice genomics and functional genomics studies. This technique is beneficial for both bridging the knowledge gap between genotype and phenotype and facilitating molecular breeding via gene design in rice. Here, we also discuss the limitation, application and future prospects of rice resequencing.

## Introduction

Rice is one of the most important staple crops worldwide as well as a model monocot used in genomics research. The world population has already exceeded seven billion and is still growing, while the amount of land suitable for agriculture is decreasing due to a variety of factors such as rapid climate change. To meet the global food demands of nine billion people by 2050, improvements in molecular genetics will be important to increase rice yield in the post-genomics era (Miura et al. [2011]; Huang et al. [2013]).

In 2002, draft genomic sequences of two rice subspecies, *O. sativa ssp*. japonica (Nipponbare) and *O. sativa ssp.* indica (93–11), were released (Yu et al. [2002]; Goff et al. [2002]) and subsequently, the International Rice Genome Sequencing Project ([2005]) completed the final genome sequence of Nipponbare. These achievements have not only greatly accelerated functional genomics research, but also provided a reference genome for resequencing rice genomes using high-throughput sequencing technologies (Gao et al. [2012]; Feuillet et al. [2011]). New sequencing technologies are also known as next-generation sequencing (NGS), which makes reference to the first generation of Sanger sequencing technology. Three mainstream NGS platforms, i.e., Illumina/Solexa, Roche/454 and ABI/SOLiD sequencing, which are known collectively as high-throughput sequencing, can generate large amounts of data in a single run and analyze more than 100 kb of DNA (Ansorge [2009]).

The advent of NGS technologies has greatly enhanced rice functional genomics and molecular breeding studies (Xie et al. [2010]; Gao et al. [2012]). The model system has been readily adapted to the new sequencing technologies, as rice is a self-fertilizing plant with the smallest completed high-quality genome among cereal crops. Furthermore, abundant diverse rice germplasm resources are available for genome-wide association studies (GWAS) and evolutionary analysis (Guo et al. [2004]; Huang et al. [2012a]). With the release of the complete bacterial artificial chromosome (BAC) physical map for the *aus* rice cultivar ‘Kasalath’ and an updated version of the whole genome sequence for the *indica* rice variety ’93-11’ (Kanamori et al. [2013]; Gao et al. [2013]), at least three references are now available together with the *japonica* ‘Nipponbare’ sequence. These advantages have enabled researchers to perform accurate alignments of short sequence reads produced by NGS with the reference genomes, as well as detailed genetic polymorphism analysis of rice in an efficient manner (Han and Huang [2013]). Using information from these studies, researchers have now succeeded in characterizing genomic variation, identifying QTLs (quantitative trait loci) by GWAS, investigating the origin of cultivated rice and performing molecular breeding studies.

In this review, we briefly review the progress of the application of rice genome resequencing to studies of genome diversification, genotyping, gene identification and cultivated rice origin. We highlight the improved parental genome sequences, effective genetic mapping and genotyping techniques involving deep resequencing of an RIL (recombinant inbred line) population of the super hybrid rice Liang-You-Pei-Jiu (LYP9) (Gao et al. [2013]). Additionally, we discuss the future of rice resequencing.

## Review

### Genetic diversity and genome variation analysis of rice germplasm

Rice has more than 100,000 accession germplasm resources including Asian cultivated rice, African cultivated rice and 22 wild rice species (The International Rice Genebank: http://irri.org/our-work/research/genetic-diversity), which are indispensable genetic resources for further improvement of cultivated rice varieties. Recently, rice molecular biology research has been involved in exploring genetic diversity and exploiting genome variation in rice germplasm. Genetic variation can be assayed using a variety of molecular markers, including structure variation (SV) markers such as insertions/deletions (InDels) and copy number variations (CNV). Resequencing can be used to identify genetic variation within a species and to assess the population structure and the pattern of linkage disequilibrium. Resequencing a key germplasm subset representing various geographically distributed populations can provide scientists with in-depth knowledge of the range of genome variation and genetic diversity within the population based on sequence databases.

Numerous diverse rice germplasm resources that have been used to detect genome variation are shown in Table 1 (Subbaiyan et al. [2012]; Jeong et al. [2013]). Kojima et al. ([2005]) detected 554 alleles from 332 accessions of cultivated rice based on a genome-wide RFLP survey, and developed a rice diversity research set of 69 accessions of germplasm, including two reference varieties, Nipponbare and Kasalath. McNally et al. ([2009]) identified 160,000 genome-wide SNPs in 20 diverse rice varieties via microarray-based resequencing and discovered their introgression patterns and pedigree relationships. Zhao et al. ([2011]) genotyped 44,100 SNP variants across 413 accessions of *O. sativa* collected from 82 countries for genetic structure analysis and cross-population-based mapping. Huang et al. ([2010]) resequenced 517 *indica* subspecies of Chinese rice landraces with approximately one-fold-coverage Illumina sequencing. A total of 3,625,200 non-redundant SNPs were identified, resulting in an average of 9.32 SNPs per kb, with 167,514 SNPs located in the coding regions of 25,409 annotated genes. A high-density SNP map and haplotype map (HapMap) of the rice genome was constructed using a novel data-imputation method. Subsequently, Huang et al. ([2012b]) extended this methodology to a larger, and more diverse, sample of 950 worldwide rice varieties, including *indica* and *japonica* subspecies. In the non-repeated regions, 4,109,366 non-singleton SNPs and 191,476 non-redundant InDels ranging from 1 bp to 376 bp in size were identified in genic regions. The authors investigated the worldwide rice population structure and constructed a neighbor-joining tree involving five divergent groups: *indica*, *aus*, temperate *japonica*, tropical *japonica* and intermediate, which were consistent with the five-distinct-groups detected by Garries et al. ([2005]). The authors resequenced an elite Japanese rice cultivar, Koshihikari, corresponding to 80.1% identity with the Nipponbare sequence, and leading to the identification of 67,051 SNPs. They also genotyped 151 representative Japanese cultivars using 1,917 SNPs to clarify the dynamics of the pedigree haplotypes (Yamamoto et al. [2010]). Based on genome-wide SNP analysis, these studies revealed relationships among landraces and modern varieties of rice, and genetic diversity that can be used for breeding programs in rice.

**Table 1:** Application and information of resequencing in rice

**Materials**	**Depth of sequencing**	**SNPs**	**Research purposes**	**References**
132 RILs of a super hybrid rice	>4×	171,847	Improving parental genome sequences	(Gao et al. [2013])
*Liang-You-Pei-Jiu*	>36× for parents		Dissecting yield-associated loci	
1083 cultivated rice*	>1 ~ 50×	7,970,359	Domestication analysis of cultivated rice	Huang et al. ([2012a])
446 wild rice			Identifying agronomic QTL	
40 cultivated rice	> 15×	6,500,000	Identifying agronomic QTL	Xu et al. ([2012])
10 wild rice			Domestication analysis	
950 cultivated rice	>1×	4,109,366	GWAS study of flowering time and grain yield traits	Huang et al. ([2012b])
517 rice landraces	>1×	3,625,200	GWAS study of 14 agronomic traits	Huang et al. ([2010])
150 RILs of Nipponbare/93-11	>20×	1,226,791	Large-scale gene discovery	Huang et al. ([2009])
			Identifying 49 QTLs for 14 agronomic traits	Wang et al. ([2011])
128 CSSLs of Nipponbare/93-11	>0.13×	7,680,000	QTL mapping for culm length	Xu et al. ([2010])
			High-throughput genotyping	
5 cultivated rice	>58×	1,154,063	Genetic diverse analysis	(Jeong et al. [2013])
A restorer line 7302R	>13×	307,627	Genetic variation identification	(Li et al. [2012])
4 other cultivated rice				
241 RILs of a hybrid rice Shanyou 63	> 0.06×	270,820	QTL detection for grains	Yu et al. ([2011])
40 RILs of Nortai/Hitomebore (bulked)	>6×	161,563	Rapid QTL mapping	Takagi et al. ([2013])
50 F_2_ lines of Dunghan Shali/Hitomebore				
781 F_2_ lines of R1128/Nipponbare	>16×	74,329	Genetic analysis for super hybrid rice	Duan et al. ([2013])
Koshihikari	15.7×	67,051	Evaluate the dynamics of the genome composition	Yamamoto et al. ([2010])

*1083 accessions of cultivated rice cultivars in the ref. of Huang et al. ([2012a]) include the 950 accessions of cultivated rice in the ref. of Huang et al. ([2012b]), which include the 517 accessions of rice landraces in the ref. of Huang et al. ([2010]).

Genome diversity has increasingly been identified in wild rice. Wing et al. ([2005]) constructed bacterial artificial chromosome and/or sequence tag connector (BAC/STC)-based physical maps of 11 wild and one cultivated rice species for alignment with the rice reference genome. Resequencing the wild species of the genus *Oryza* has enormous potential in identifying approaches to increase agricultural productivity of the cultivated rice species *O. sativa* and *O. glaberrima*. Xu et al. ([2012]) directly resequenced 50 accessions of cultivated and wild rice, and identified genome-wide variation patterns including the identification of more than 6.5 million high-quality SNPs, 808,000 InDels, 94,700 SVs (>100 bp) and 1,676 CNVs. In another study, 66 accessions from three taxa (22 each from *O. sativa indica*, *O. sativa japonica* and *O. rufipogon*) were chosen for whole genome sequencing (He et al. [2011]). Huang et al. ([2012a]) also generated genome sequences from 446 geographically diverse accessions of the wild rice species *O. rufipogon*, the immediate ancestral progenitor of cultivated rice, and from 1,083 cultivated *indica* and *japonica* varieties, to construct a comprehensive map of rice genome variation. A total of 7,970,359 non-singleton SNPs were identified from the 1,529 available rice genome sequences, the ancestral alleles of 9.3% of which are identical to those of *O. rufipogon*. These genotype data and population genetics analyses provide insights into the relationships between rice diversity and domestication processes.

The NGS strategy also provides new opportunities for RNA sequence diversity analysis or epigenomic studies in rice. The functional complexity of the rice transcriptome and its contribution to phenotype remains to be fully elucidated. Gene expression microarrays have been used traditionally for high-throughput measurements of gene expression levels (Jiao et al. [2005]; Furutani et al. [2006]; Li et al. [2006b]; Satoh et al. [2007]). Recently, with the development of the next-generation high-throughput DNA sequencing technologies, RNA-seq has shown advantages over microarrays by allowing accurate, efficient and reproducible estimations of transcript abundance of either known or unknown transcripts with a larger dynamic range using less RNA sample (Wilhelm et al. [2008]; Fullwood et al. [2009]). Furthermore, RNA-seq can detect genes expressed at low levels and refine the structure of transcripts (Wang et al. [2009]; Wilhelm and Landry [2009]). Using high-throughput paired-end RNA-seq, a substantial number of novel transcripts and exons have been detected, and a far greater amount of alternative splicing has been identified that was shown previously (Zhang et al. [2010]). The updated research focused on comparative analyses of the epigenome and comprehensive analyses of the eQTL (He et al. [2010]; Lu et al. [2010]). Additionally, NGS strategy has been applied recently in massive program of sequencing of small RNA populations from different rice tissues. Jeong et al. ([2011]) also identified 76 new rice miRNAs that play critical roles in a variety of developmental processes. Wang et al. ([2012]) sequenced an accession of *O. rufipogon* to *c.* 55× coverage, identified miRNAs using small RNAs generated from three different tissues of *O. rufipogon* and identified miRNA targets in *O. rufipogon* by degradome sequencing. The authors found that rice miRNA genes have experienced a complex evolutionary process during domestication. This plethora of genetic diversity RNA data is also an important genetic resource for rice breeding.

### Evolution analysis based on resequencing

Cultivated rice is thought to have been domesticated from wild rice thousands of years ago. However, the evolutionary origins and domestication processes of cultivated rice have long been debated. A wide range of genetic and archeological studies have been carried out to investigate rice phylogenetics and the demographic history of rice domestication. Some population genetics studies have indicated that *indica* and *japonica* originated independently, and some demographic analyses have suggested that domesticated rice has a single origin (Kovach et al. [2007]; Molina et al. [2011]). With the development of NGS technologies, analysis of the global diversity of rice germplasm and sequencing-based genome variation analysis can provide in-depth insights into rice domestication.

Recently, Huang et al. ([2012a]) systematically constructed a comprehensive map of rice genome variation with 6,119,311 SNPs using the 1,529 rice genome sequences. Phylogenetic tree analysis indicated that *O. sativa* and japonica are descended from *O. rufipog*on*-I* and *Or-III* (the *O. rufipogon* species were classified into three types, i.e., *Or-1*, Or*-II* and *Or-III*), respectively. A total of 213,188 *indica-japonica*-differentiated SNPs were found, and only 9,595 SNPs were fixed between *O. rufipogon* and *O. sativa*. The differentiation was enhanced during domestication, with the divergence index Fst expanding from 0.18 in *O. rufipogon* to 0.55 in *O. sativa*. The level of genetic differentiation between indica and *Or-I* was modest (Fst = 0.17), and that is 0.36 in japonica. The authors found that only about 33% of the genetic diversity of *Or-III* persisted in japonica. Moreover, indica contains approximately 75% of the genetic diversity observed in *Or-I*. In a search for signatures of selection, the authors identified 32 selective sweeps in the rice genome and 55 domestication loci by searching for signatures of domestication using an integrated genomics approach. The authors detected a series of gene introgression events. The most well-characterized domestication genes, such as *Bh4, PROG1, sh4, qSW5* and *OsCl*, were among the 55 loci detected in the total population (Zhu et al. [2011]; Shomura et al. [2008]; Saitoh et al. [2004]). However, an additional three genes, *qSH1*, *Waxy* and *Rc*, were detected only in the japonica panel. SNP-based phylogenetic tree analysis showed that the middle region of the Pearl River district in Guangxi Province, southern China, is probably the origin of development of cultivated rice. It can be speculated that japonica was possibly first domesticated and then crossed with local wild rice in Southeast Asia to generate *indica*.

Also Xu et al. ([2012]), used a haplotype map of 6.5 million SNPs from 50 rice genome sequences to identify thousands of genes with significantly lower diversity in cultivated rice than that in wild rice; these genes represent candidate regions selected during domestication. A total of 73 candidate genes that underwent sweeps in both japonica and indica were identified by comparing polymorphism levels in cultivated and wild species. These polymorphism levels were calculated by the reduction of diversity values (*ROD =* 1 − π_*cul*_/π_*wild*_), based on the ratio of the diversity in cultivated rice to the diversity in wild rice. Two well-known rice domestication genes, *prog1* (Jin et al. [2008]; Tan et al. [2008]) and *sh4* (Li et al. [2006a]), were successfully identified in the putative artificial selection gene set. Gene families related to morphology, growth and transcriptional regulation were enriched among many of the candidate genes. All of these functionally uncharacterized or unknown candidate genes related to artificial selection provide useful guidance for rapidly identifying genes of agronomic significance in rice. Population structure and phylogenetic analyses not only support the hypothesis that japonica and indica were domesticated independently, but they also suggest that japonica was domesticated from the Chinese strain of *Oryza rufipogon*. The data generated in this study provide a valuable resource for rice improvement.

### QTL/gene identification by sequencing-based genotyping in rice

The majority of important agronomic traits in rice are controlled by multiple genes (namely, QTLs). QTL mapping is important for understanding the mechanisms underlying complex agronomic traits via genotyping and phenotyping of a classical population (RIL, DH or BCF_2_) derived from a cross between two cultivars. Conventional QTL mapping is a powerful method for QTL identification and cloning; however, compared with resequencing-based linkage maps or bin maps, it is generally regarded as a time-consuming and laborious process because lower resolution linkage maps are constructed with low-throughput molecular markers (usually simple sequence repeats, SSR) (Huang et al. [2012b]). Therefore, sequencing-based genotyping provides a more powerful tool for large-scale QTL/gene discovery.

Gao et al. ([2013]) resequenced and genotyped 132 core RILs derived from a cross between the two rice varieties PA64s and 93–11 to construct a SNP-based ultra-high-density linkage map using the NGS method. A total of 43 yield-associated QTLs, including 20 newly identified QTLs, were mapped using a 3,524-SNPs linkage map. Ten QTLs were further mapped using a larger RIL population and two QTLs, *qSN8* and *qSPB1*, were delimited to regions each covering one candidate yield-related gene, *DTH8* and *LAX1* (Table 2). This precise QTL mapping from core to larger RIL populations using a sequencing-based approach will greatly facilitate QTL cloning and molecular breeding.

**Table 2:** Key allelic loci fine-mapped using the NGS method in rice

**Trait**	**QTL**	**Chromosome**	**Mapping materials**	**Reference**	**Allelic loci**
Tiller angle	qTA-9	9	Nipponbare/93-11	Wang et al. ([2011])	*TAC1* (Yu et al. [2007])
Plant height	qPH-1	1	Nipponbare/93-11	Wang et al. ([2011])	*SD1* (Sasaki et al. [2002])
Flag leaf width	qFLW-4	4	Nipponbare/93-11	Wang et al. ([2011])	*NAL1* (Qi et al. [2008])
Grain length	qGL-3	3	Nipponbare/93-11; PA64s/93-11; Zhenshan 97/Minghui 63	Wang et al. ([2011]), (Gao et al. [2013]), Yu et al. ([2011])	*GS3* (Fan et al. [2006])
Grain width	qGW-5	5	Nipponbare/93-11	Wang et al. ([2011])	*qSW5* (Shomura et al. [2008])
Heading date	qHD8	8	PA64s/93-11	(Gao et al. [2013])	*DTH8* (Wei et al. [2010])
Plant height	qPH5	5	PA64s/93-11	(Gao et al. [2013])	*EUI1* (Luo et al. [2006])
Plant height	qPH12	12	PA64s/93-11	(Gao et al. [2013])	*NRL1* (Hu et al. [2010])
Effective tiller number	qETN4	4	PA64s/93-11	(Gao et al. [2013])	*HTD1* (Zou et al. [2005])
Secondary panicle branch No.	qSPB1	1	PA64s/93-11	(Gao et al. [2013])	*LAX1* (Komatsu et al. [2001])
Seed set	qSS12	12	PA64s/93-11	(Gao et al. [2013])	*P/TMS12-1* (Zhou et al. [2012])
Hull color	Domesticaton sweeps	4	Natural population	Huang et al. ([2012a])	*Bh4* (Zhu et al. [2011])
Tiller angle	Domesticaton sweeps	7	Natural population	Huang et al. ([2012a]), Xu et al. ([2012])	*PROG1* (Jin et al. [2008]; Tan et al. [2008])
Seed shattering	Domesticaton sweeps	4	Natural population	Huang et al. ([2012a])	*Sh4* (Li et al. [2006a])
Grain width	Domesticaton sweeps	5	Natural population	Huang et al. ([2012a])	*qSW5* (Shomura et al. [2008])
Leaf sheath color & apiculus color	Domesticaton sweeps	6	Natural population	Huang et al. ([2012a])	*OsC1* (Saitoh et al. [2004])
Seed shattering	Domesticaton sweeps	1	Natural japonica population	Huang et al. ([2012a])	*qSH1* (Konishi et al. [2006])
Grain quality	Domesticaton sweeps	6	Natural japonica population	Huang et al. ([2012a])	*Waxy* (Wang et al. [1995])
Pericarp color	Domesticaton sweeps	7	Natural japonica population	Huang et al. ([2012a])	*Rc* (Sweeney et al. [2006])
Grain width	gw5, kgw5	5	Zhenshan 97/Minghui 63	Yu et al. ([2011])	*GW5/qSW5* (Shomura et al. [2008])
Pigmentation	qPIG6	6	Zhenshan 97/Minghui 63	Yu et al. ([2011])	*OsC1* (Saitoh et al. [2004])
Gelatinization temperature	qGT6	6	517 rice landraces	Huang et al. ([2010])	*ALK* (Gao et al. [2003])
Plant height	qPH1-3	1	R1128/Nipponbare	Duan et al. ([2013])	*Sd1* (Sasaki et al. [2002])
Heading date	qPBN6-2	6	R1128/Nipponbare	Duan et al. ([2013])	*Hd1* (Yano et al. [2000])
Early heading date	qPBN10-1	10	R1128/Nipponbare	Duan et al. ([2013])	*Ehd1* (Doi et al. [2004])
Grain number 1	qGN1-1	1	R1128/Nipponbare	Duan et al. ([2013])	*Gn1* (Ashikari et al. [2005])
Ideal plant architecture	qPL8-1	8	R1128/Nipponbare	Duan et al. ([2013])	*IPA1* (Jiao et al. [2010])

Wang et al. ([2011]) resequenced 150 RILs of 93-11/Nipponbare to construct SNP-based ultra-high-density linkage maps and identified 49 QTLs for 14 agronomic traits. Xu et al. ([2010]) resequenced 128 CSSLs of 93-11/Nipponbare to construct a bin map. Nine QTLs for culm length were fine-mapped and one QTL was located in a 791,655-bp region containing the rice “green revolution” gene *sd1*. Xie et al. ([2010]) sequenced 238 RILs of Zhenshan 97/Minghui 63 and constructed a 209,240-SNPs genetic map. Using the SNP bin map, Yu et al. ([2011]) identified 22 QTLs for four yield traits. The mapping interval of *GS3* for grain length was narrowed down from a 6.0-Mb region in the RFLP/SSR genetic map to a 197-kb region. This indicated that the high-density SNP bin map could improve the power of QTL detection. Compared with the PCR-based marker method, the sequencing-based method was faster and more precise for the determination of the recombination breakpoint, achieving a resolution of 40 kb recombinant block on average (Wang et al. [2011]).

Sequencing-based GWAS has recently been applied as a high-throughput QTL mapping method to dissect agronomic traits in a variety of rice germplasm resources. Han and Huang et al. ([2013]) published a brief scheme from a typical sequence-based GWAS involving the identification of high-quality haplotypes to associate molecular markers with phenotypes accurately. QTL mapping by GWAS in rice germplasm can be used as a complementary strategy for classical biparental cross-mapping of dissecting complex traits, but it seems to be effective only for genes with large effects.

Using the GWAS method, Huang et al. ([2010]) identified 49 QTLs for 14 agronomic traits via resequencing 517 rice landraces. Six loci were confirmed to be close to previously identified genes. They later used 950 worldwide rice varieties and identified 32 new loci associated with flowering time and 10 with grain-related traits, indicating that the use of a larger sample size increases the power to detect trait-associated QTLs using GWAS (Huang et al. [2012b]), Zhao et al. ([2011]) identified dozens of common variants influencing 34 complex traits via resequencing 413 diverse accessions of *O. sativa*. These GWAS research platforms in rice directly link molecular variation in genes and metabolic pathways with the germplasm resources, accelerating varietal development and crop improvement.

Large-scale genome sequencing can also be used for rapid mapping of rare and spontaneous mutations using resequencing technologies. The SHOREmap pipeline developed for this purpose has been incorporated into several modules and performs various functions, from mapping to de novo marker identification through deep sequencing, as well as annotation of candidate mutations in Arabidopsis (Schneeberger and Weigel [2011]). MutMap (mutation map), or Next-Generation Mapping (NGM), is a method for rapid isolation of mutant genes based on whole genome resequencing of small pooled DNA from an F_2_ or F_3_ segregating population (Austin et al. [2011]; Abe et al. [2012a]). Yang ([2013]) used bulked segregant analysis (BSA), combined with NGS (referred to as NGS-BSA) to identified six QTLs for cold tolerance at the seedling stage via resequencing DNA pools from 385 extremely tolerant F_3_ individuals of Nipponbare/LPBG. QTL-seq is another method for rapid identification of QTLs by resequencing RILs or F_2_ genetic populations composed of 20–50 individuals per population showing extreme opposites in trait values for a given phenotype in segregating progeny. Takagi et al. ([2013]) identified two QTLs for seedling vigor using 531 F_2_ individuals of Dunghan Shali/Hitomebore. Two loci were confirmed to be close to the previously reported QTLs *qPHS3-2S* and *qPHS-1* (Yano et al. [2012]; Abe et al. [2012b]). QTL-seq can generally be applied in population genomics studies to rapidly identify genomic regions that have undergone artificial or natural selective sweeps.

### Improvement of biparental genome sequences of a hybrid rice

Hybrid rice breeding has great potential for improving rice yield. Our research group has resequenced 132 RILs derived from a cross between PA64s and 93–11, the parents of the pioneer super hybrid rice LYP9, together with their parents using Solexa sequencing technologies. We generated 244 GB of raw data, with approximately four-fold depth per RIL, 48-fold depth for PA64s and 36-fold depth for 93–11. A linkage map was constructed with an average interval of approximately 0.392 cM between recombinant blocks (Gao et al. [2013]).

Based on the constructed map of the graphic genotypes, we distinguished the reads from 132 LYP9 RILs to fill in the remaining gaps in the PA64s genome sequence. A total of 29.3 Mb new sequences were used to fill in the gaps. In the non-repeated regions, 36,033 loci with homozygous genotypes were identified as single base errors. Using the Sanger strategy, the genome sequence of PA64s now comprises 382 Mb.

**Figure 1:**
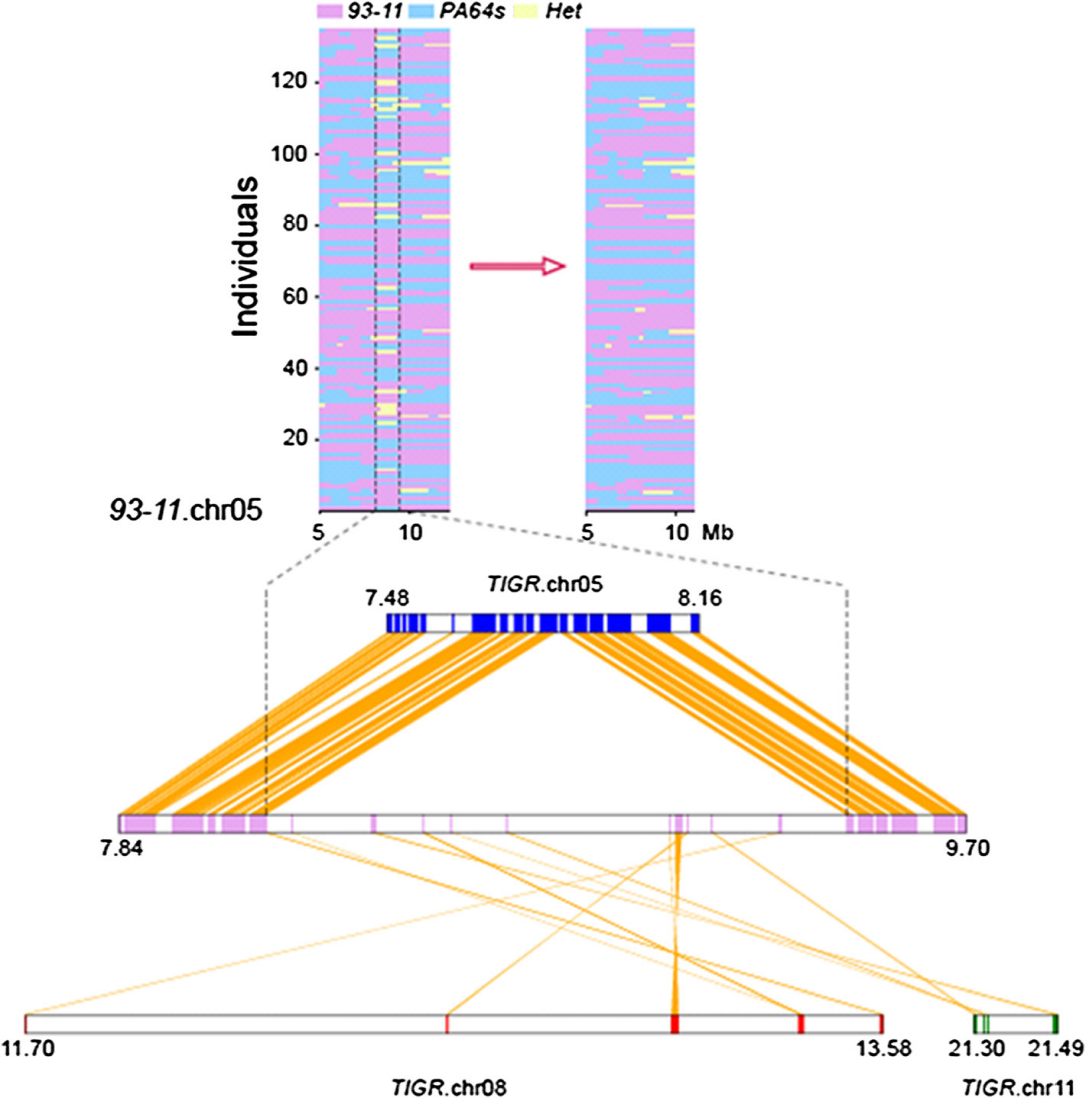
**An example of falsely anchored scaffolds revealed by linkage map and syntenic analysis.** The region of falsely anchored scaffolds disturbed the normal linkage relationships in the graphic map of the genotypes and was not supported by normal syntenic analysis with the reference.

Similarly, we also updated the published 93–11 genome sequences, which comprise 423 Mb, including 369.8 Mb quality sequences located on chromosomes, filling in approximately 3.8 Mb of new sequences to cover 1,493 gaps. Using the linkage map obtained from the RILs, we realigned the PDRs (parental derived reads) to the 93–11 genome sequence and corrected 62,650 SNP errors (Figure 1). The high-quality genome sequences of PA64s and 93–11 were improved by RIL linkage mapping, providing the basis for detailed genetic analysis. Therefore, resequencing a segregating population can be used to improve multiple reference genome sequences. The improved sequence information, which was uploaded to the rice genome website (http://rice.genomics.org.cn/rice/), may be beneficial for improving super hybrid rice (Gao et al. [2013]).

### Future perspectives

The birth of NGS technologies is a landmark event in functional genomics, creating a new era of rice resequencing in a highly accelerated manner based on the high-quality rice reference genomes. It provides the large amount of genome sequencing data available in rice, including more than 1,500 one-fold rice genome sequences from natural populations and thousands of low-fold genome sequences from various rice lines of crossed-based genetic populations of DH, RILs F_2_ or ILs (introgression lines) (Tables 1 and 2), even the increasing reference genome sequences such as Nipponbare, 9311, and Kasalath (Kanamori et al. [2013]). The genome sequences of PA64s and GLA4 are further improving. Furthermore, increasing numbers of rice germplasm resources and genetic materials are being resequenced; for example, the International Rice Research Institute (IRRI), Chinese Academy of Agricultural Sciences (CAAS) and Beijing Genomics Institute (BGI) –Shenzhen, have collaboratively been resequencing 3,000 accessions of global rice germplasm from the IRRI genebank since 2010. The increasing amount of sequenced genome information has been applied broadly in analyses of genetic diversity, genome variation, RNA sequences and domestication processes, even in cultivars improvement. The resequencing-based method shows a series of obvious advantages; it is approximately 20-times faster in terms of data collection and 35-times more precise in detecting recombination breakpoints, compared to genetic mapping with PCR-based markers (Huang et al. [2009]). However, one of the major drawbacks that limit the use of NGS technology, especially in de novo sequencing, is the short sequencing read lengths. Owing to significant inter- and intra-species chromosomal structural changes induced in rice by InDels, duplications, inversions, translocations and transpositions (Wu et al. [2009]; Hurwitz et al. [2010]; Lin et al. [2012]), assembly and mapping of NGS short-read sequences is complex and relatively difficult. High-quality sequencing approaches have been suggested in conjunction with high-throughput sequencing for comparative genomics analyses and genome evolution studies (Alkan et al. [2011]). A technology which combines the massive throughput of the NGS with the long read lengths achieved by electrophoresis-based Sanger sequencing, would enable rapid, high-quality production of de novo genome sequences (Hert et al. [2008]). Future directions in the field of DNA sequencing are the ability to use individual molecules without any library preparation or amplification, the identification of specific nucleotide modifications, and generation of longer sequence reads (Kircher and Kelso [2010]). Therefore, it is necessary to develop multiple sequencing approaches and platforms, including the third-generation long read technologies, high-quality long-insert clones and new assembly algorithms.

Despite the advantages of efficiency in resequencing, the availability of a series of reasonable and adequate genetic resources is a prerequisite for the implementation of this technology. More than 100,000 accessions of diverse rice germplasm in global genebanks are available, but in fact, genetic diversity in modern rice cultivars has become increasingly narrow. In order to enhance the identification and use of the genetic diversity in rice, Frankel ([1984]) proposed the development of “core collections” for effective use of these germplasm. Subsequently, a series of “core collections” has been developed based on agro-morphological or biochemical traits and ecogeographical information as well as molecular markers and genome information (Kojima et al. [2005]; Huang et al. [2009]). Kojima et al. ([2005]) developed a rice diversity research set of germplasm (RDRS) from the NIAS (National Institute of Agrobiological Sciences, Japan) Genebank for in-depth genetic diversity analysis via development of genetic materials, such as substitute backcrossed lines or chromosome introgression lines. Yamamoto et al. ([2010]) clarified the dynamics of the pedigree haplotypes using 151 representative Japanese cultivars and 1,917 polymorphism SNPs between Nipponbare and Koshihikari. This information can be used to predict particular haplotypes associated with desirable phenotypes during the Japanese rice breeding process, and help in using such haplotype blocks for rice improvement. Sampling and population sizes directly affect genome variation analysis; therefore, Huang et al. ([2012a]) used more germplasm resources and a larger natural population to detect genome variation. Using this approach, at least 7,970,359 SNPs were identified from 1,529 genome sequences that were successfully used in the search for signatures of selection. We sequenced *LYP9* RILs, and established an ideal platform for molecular breeding. Accumulated data on the various traits and on genome polymorphism in the collection will be beneficial for the construction of a genotype-phenotype database and will enhance the efficient use of rice genetic resources for the development of new cultivars.

Broadening genetic diversity in rice is one of the most important breeding measures required for overcoming the bottleneck in increasing yield. Discovering valuable genes and alleles from the global rice germplasm demands a long-term collective effort to increasing genetic diversity. The currently available genome information indicates that much more natural allelic variations are present in wild rice than in domesticated rice. Breeders have mainly utilized intersubspecies heterosis and elite genes of wild rice germplasm to broaden genetic diversity, such as hybrid rice sterility lines Zhenshan97A with the wild rice CMS-DA (cytoplasmic male sterility) gene and the *indica* sterile line PA64s on a *japonica* background. The functional allelic loci obtained from sequencing-based GWAS mapping have greatly accelerated diverse germplasm mining and molecular breeding. More than 700 cloned rice genes and the sequencing-based GWAS loci will help fulfill the ultimate goal of determining the function of every gene in the rice genome in future through a highly coordinated effort facilitated by the International Rice Functional Genomics Project (IRFGP) (Zhang et al. [2008]; Ikeda et al. [2013]). The identification of the functional genes in cultivated rice and the high degree of natural allelic variation in wild rice will be beneficial for developing elite super rice varieties such as new idiotype super rice with *indica-japonica* heterosis (Liu et al. [2012]; Guo and Ye [2014]) or green super rice (Zhang [2007]) through genome selection or molecular breeding via gene design.

## Conclusion

In this review, we summarize the current status of whole genome resequencing of diverse germplasm resources in rice using NGS technology. More than 1,500 accessions of rice germplasm resources from natural populations and thousands of various rice lines of biparental populations have been resequenced, and effectively utilized in evolutionary analysis, rice genomics and functional genomics studies. We discuss the limitations, application and future prospects of rice resequencing.

## Abbreviations

NGS: Next-generation sequencing

GWAS: Genome-wide association studies

SNP: Single ncleotide polymorphisms

BAC: Bacterial artificial chromosome

SV: Structure variation

InDels: Insertions/deletions

CNV: Copy number variations

## Competing interests

The authors declare that they have no competing interests.

## Authors’ contributions

LG and ZG performed the analysis, prepared the tables and figures. LG and QQ wrote the paper. All authors read and approve the final manuscript.
